# Rare delayed cardiac tamponade in a pig after cardiac surgery

**DOI:** 10.1002/vms3.892

**Published:** 2022-08-03

**Authors:** Ke Li, Ana Maria Segura, Junping Sun, Qi Chen, Jie Cheng, Emerson C. Perin, Abdelmotagaly Elgalad

**Affiliations:** ^1^ Center for Preclinical Surgical and Interventional Research Texas Heart Institute Houston Texas USA; ^2^ Department of Cardiovascular Pathology Texas Heart Institute Houston Texas USA; ^3^ Electrophysiology Basic Research Texas Heart Institute Houston Texas USA; ^4^ Center for Clinical Research Texas Heart Institute Houston Texas USA

**Keywords:** animal models, cardiac tamponade, pericardial effusion, postoperative complication

## Abstract

**Objective:**

Delayed cardiac tamponade, a life‐threatening complication of pericardial effusion in humans, has rarely been described in large animal models. We report here a pig with cardiac tamponade that developed 29 days after cardiac surgery.

**Study Design:**

Case report.

**Animals:**

One 45‐kg domestic pig.

**Methods:**

Open‐chest surgery was performed on a pig to induce chronic heart failure. At 15 days after surgery, the pig's breathing appeared laboured; induced heart failure was considered the cause. Routine heart failure medications were administered.

**Results:**

On day 28, the pig's status deteriorated. On day 29, echocardiography performed just before the pig's death showed a large pericardial effusion, mainly in the lateral and anterior walls of the right heart, with several fibre exudation bands. The right heart was severely compressed with an extremely small right ventricle. An emergency sternotomy was unsuccessful. Pathologic examination showed a severely thickened, fibrous pericardium. The pericardial sac was distended (up to 4.5 cm) and was full of dark brown, soft, friable material. Epicardial haemorrhage with a fresh, organised thrombus was noted in the pericardium.

**Conclusion:**

Delayed tamponade occurring at least 15 days after open‐chest surgery is easy to misdiagnose or overlook in large animal models where attention is often focused on primary pathological model changes. To decrease mortality in animal models, researchers should be aware of potential complications and use the same level of follow‐up monitoring of large animals as in clinical care.

## Graphical abstract

In this report, we describe the development of delayed cardiac tamponade in a pig 29 days after cardiac surgery. This life‐threatening complication has rarely been described in large animal models and is easy to misdiagnose or overlook because attention is often focused on primary pathological model changes. To decrease mortality in animal models, researchers should be aware of potential complications and use the same level of follow‐up monitoring of large animals as in clinical care.

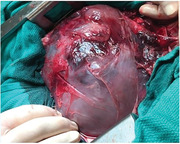

## 1 INTRODUCTION

In the clinical setting, the incidence of pericardial effusion after cardiovascular surgery is high (ranging from 50% to 85%) and peaks at the end of the first postoperative week (Ikaheimo et al., 1988). Pericardial effusion after postoperative day 15 is less common and is associated with a higher incidence of cardiac tamponade, which is then called delayed or late tamponade (Meurin et al., 2004). Cardiac tamponade results in circulatory failure secondary to compression of the cardiac cavity by the accumulating pericardial effusion. It is a life‐threatening complication of pericardial effusion in humans but is rarely described in large animal models. In recent years, more pre‐clinical cardiovascular research models are being developed in large animals. Here, we report a case of cardiac tamponade in a pig 29 days after cardiac surgery.

## 2 MATERIALS AND METHODS

To develop a chronic ischemic heart failure model, we performed open‐chest surgery on a domestic pig (45 kg, 4 months old). All procedures used in this study were approved by our institute's animal care and use committee. On day 0, the pig underwent open‐chest surgery under general anaesthesia. To open the pericardium, we performed a left lateral thoracotomy through the fourth intercostal space. We inserted a catheter in the right femoral artery, and using fluoroscopic guidance via left coronary angiography, we placed 2 ameroid constrictors (Research Instrument SW, Escondido, CA) in the proximal left circumflex artery (COR‐2.0‐SS, diameter = 2.0 mm) and the left proximal marginal artery (COR‐1.50‐SS, diameter = 1.5 mm). The pericardium was kept open after the constrictors were appropriately placed and installed. The ribs were reopposed, and the muscles and skin were closed in layers. A chest tube was placed before chest closure and kept in place for 2 h to monitor bleeding. After confirming there was no bleeding, we removed the chest tube. Then, the pig was transferred to the ICU and, once stable, was moved to a normal‐care cage.

## 3 RESULTS

On day 15, the pig's heart rate increased during sleep from 100 to 130 beats per minute, and the respiratory rate also increased. The pig appeared to be in distress. We ascribed these symptoms to the successful induction of heart failure. Sacubitril/valsartan (24/26 mg, orally, twice daily; Novartis Pharmaceuticals Corporation, East Hanover, NJ) and nebivolol (5 mg, orally, once daily, AbbVie, Chicago, IL) were given to mitigate the heart failure symptoms.

**FIGURE 1:**
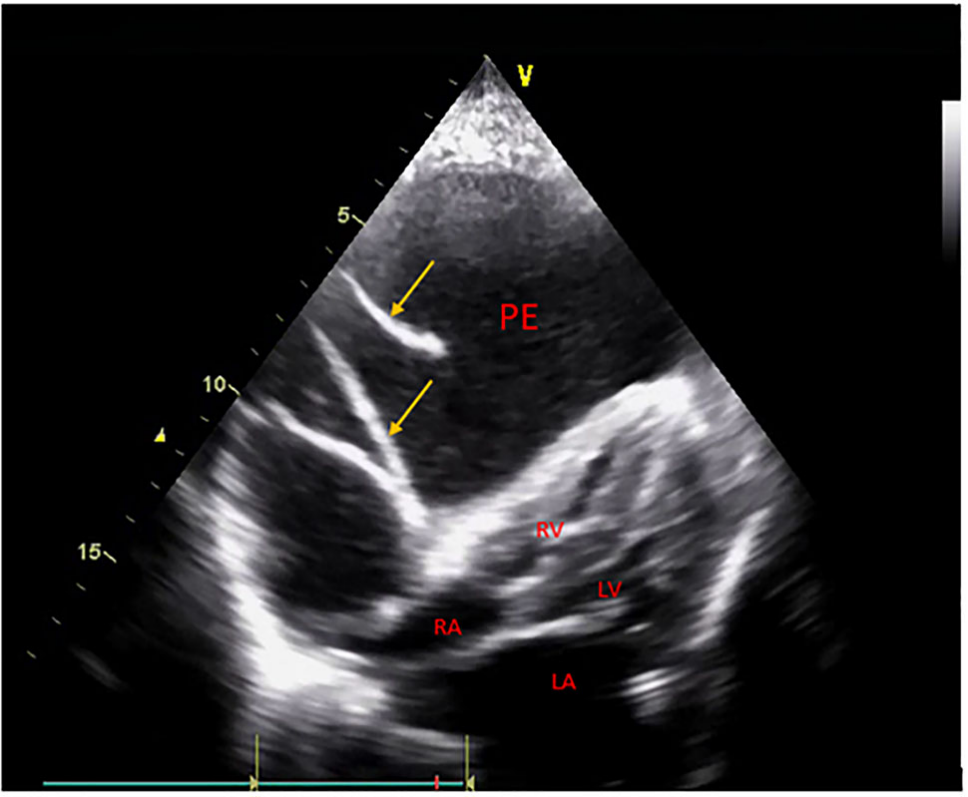
Echocardiogram showing a compressed heart (especially the right ventricle) due to a large pericardial effusion. Yellow arrows indicate fibre exudation bands. PE, pericardial effusion; RV, right ventricle; RA, right atrium; LV, left ventricle; LA, left atrium

However, the pig's status deteriorated over time. On day 29, emergency echocardiography was performed. A large pericardial effusion was seen, mainly in the lateral and anterior walls of the right heart, with several fibrinous exudation bands (Figure 1). As a result of the effusion, the right heart was compressed severely, and the right ventricular cavity was extremely small. The pig deteriorated rapidly after the echocardiography procedure, and emergency sternotomy was unsuccessful in relieving the tamponade.

**FIGURE 2:**
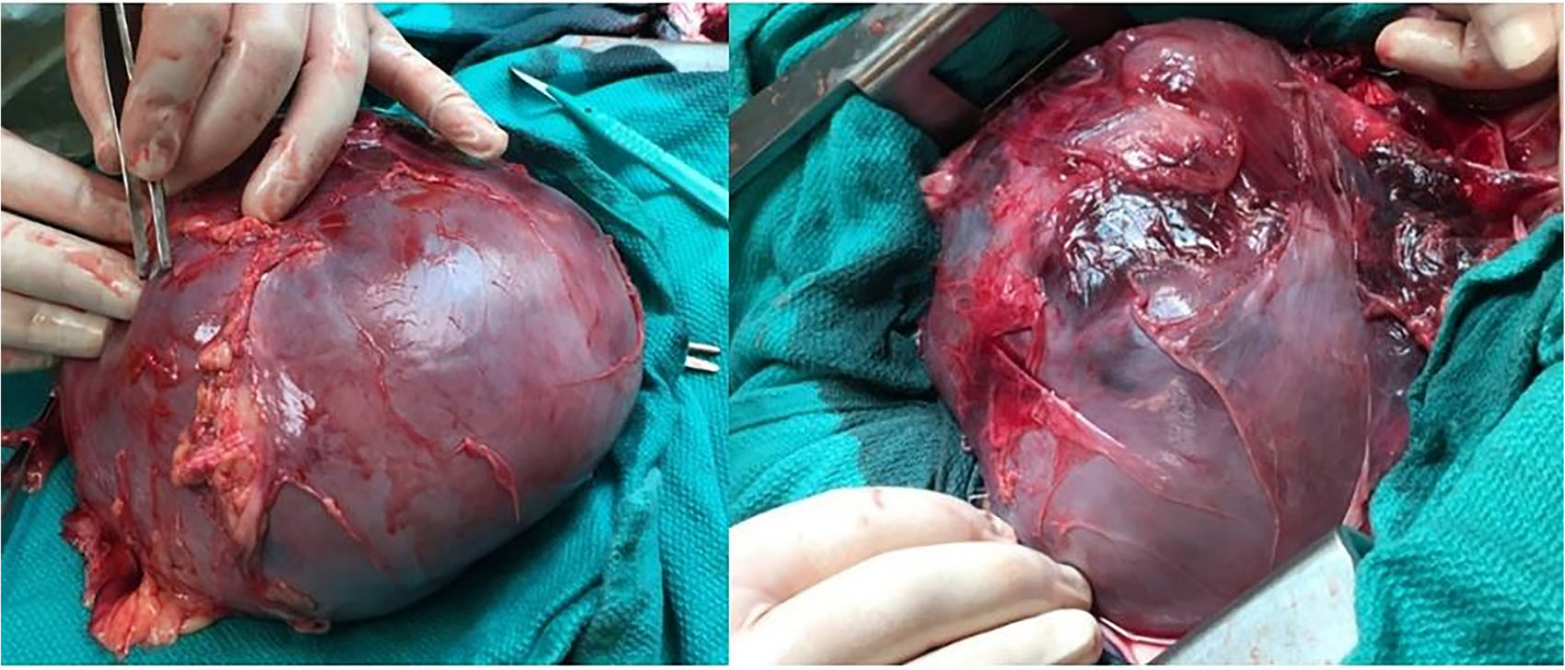
Image showing an enlarged heart with extensive haemorrhage in the pericardium

**FIGURE 3:**
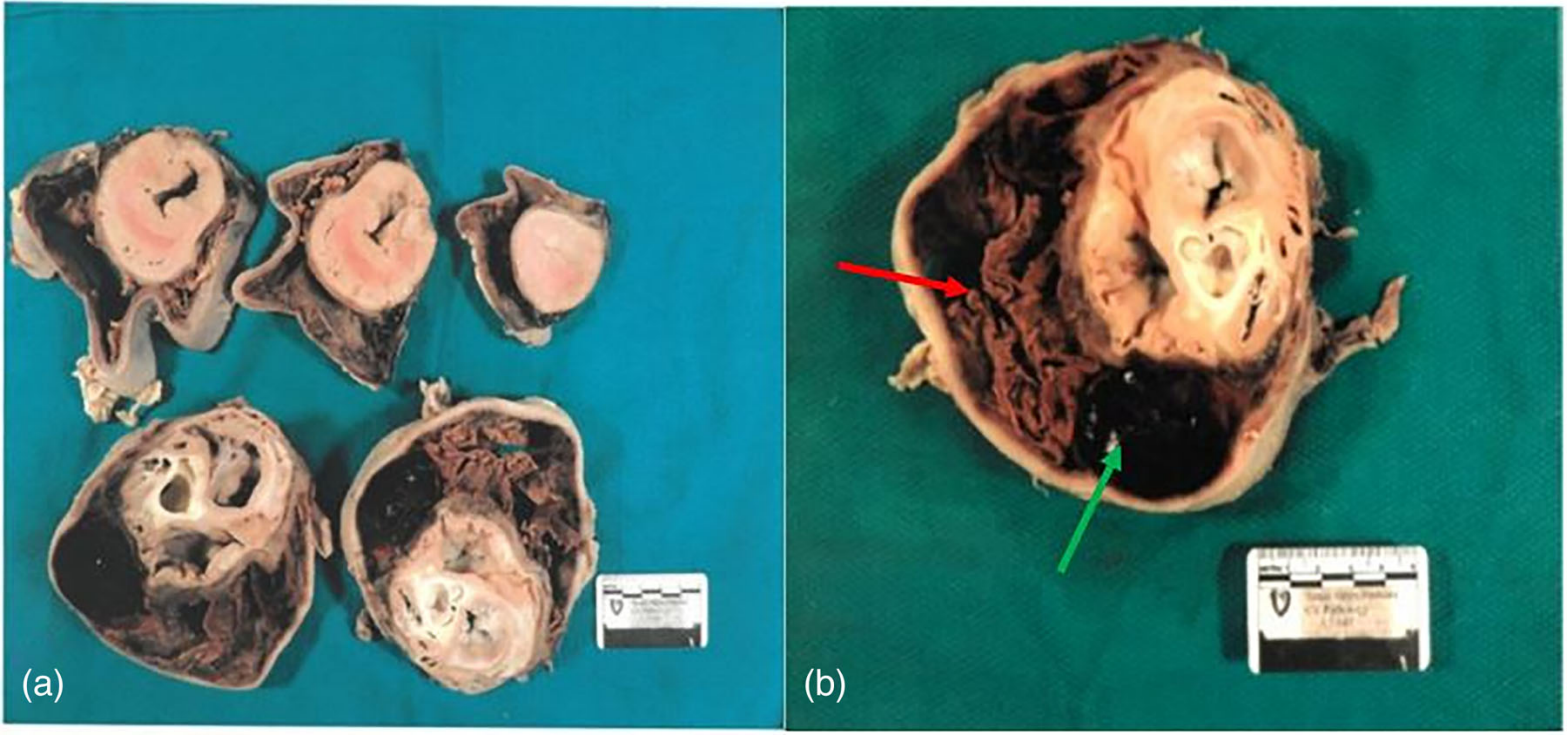
(a) Multiple heart sections showing the pericardial sac was enlarged from the apex to the base. (b) Pathological examination of the enlarged pericardial sac showing a significantly thickened pericardium and an organised thrombus (red arrow) and fresh thrombus (green arrow) inside the sac

We harvested the heart, which was enlarged with extensive pericardial haemorrhage and dark brown discoloration (Figure 2). On pathologic examination, the pericardium was severely thickened (up to 0.5 cm in thickness) and fibrous. The pericardial sac was markedly distended (Figure 3a), measuring up to 4.5 cm, and was full of dark brown, soft, friable material. Epicardial haemorrhage was seen with focal extension to the anterior walls of the left and right ventricles. An organising thrombus with fibrin deposition was attached to the pericardium and extended from the pericardial wall to the ventricular surface (Figure 3b). A fresh thrombus was seen on the surface of the right ventricular wall and extended to the pericardium (Figure 3b).

## 4 DISCUSSION

In this report, we describe a case of cardiac tamponade in a pig after cardiac surgery. Our report is a typical example of late tamponade, with symptoms starting on day 15 and tamponade occurring on day 29 after surgery. In humans, few studies mention the exact incidence of late tamponade. In a report from the 1970s, late tamponade was described as being rare, occurring in 0.5%–2.6% of patients undergoing cardiac surgery (Merrill et al., 1976). However, late tamponade is considered a significant postoperative complication of cardiac surgery because it can develop silently in the absence of obvious clinical signs. Thus, it may be easily missed and, without early diagnosis and treatment, may have a 30‐day mortality rate as high as 3% (Carmona et al., 2012, Harskamp & Meuzelaar, 2010). Because fatal events due to tamponade are rare with timely intervention, delayed tamponade in large animals deserves more attention.

Although the exact pathology of late cardiac tamponade is not fully understood (James et al., 2013), its risk factors have been discussed. These risk factors include valve replacement surgery, anticoagulation therapy, and higher severity of pericardial effusion on postoperative echocardiography (Meurin et al., 2004). In a study of 141 cardiac surgery patients who received either anticoagulation therapy (warfarin or heparin) or antithrombotic therapy (aspirin plus dipyridamole) after surgery, 12 patients developed late tamponade. All 12 had received anticoagulation therapy (Malouf et al., 1993). Similar findings were reported in a recent study (You et al., 2016). In our case, the pig had been given 162.5 mg of aspirin daily after surgery. Although we did see haemorrhagic effusion, it is less likely that an aspirin‐only regimen contributed to the late tamponade.

In studying the severity of pericardial effusion on postoperative echocardiography in the development of late tamponade, Meurin et al. reported that a loculated effusion more than 10 mm or the presence of circumferential effusion increased the possibility of late tamponade (Meurin et al., 2004). In our case, the pericardial sac was extremely distended, indicating a large effusion. It is likely that the slow progress of bleeding into the pericardial sac led to a slow rise in pericardial pressure, which eventually reached a level sufficient to decompensate heart function (Ivens et al., 2007). Although the reason for the persistent effusion is unknown, periodic transthoracic echocardiography after surgery could be used for early detection and classifying the severity of the effusion, which would help prevent tamponade. In a study of 156 patients undergoing valve surgery, the most powerful predictor of cardiac tamponade was the finding of any amount of pericardial effusion on the first postoperative transthoracic echocardiogram, which was usually performed about 5 days after surgery (You et al., 2016). Unfortunately, in our case, echocardiography was not scheduled after surgery.

Without echocardiography, there are no other specific methods to monitor pericardial effusion or prevent cardiac tamponade in large animals. Detecting “Beck's triad” of hypotension, jugular venous distension, and muffled heart sounds, which is the classic presentation of cardiac tamponade (Sternbach, 1988), is challenging in large animals. Blood pressure monitoring in animals is invasive and is not routinely used after surgery, making it difficult to observe hypotension. In addition, heart sounds may be distant due to anatomical variability in chest wall anatomy in animals, making heart sounds unreliable. Because animals cannot express discomfort early, investigators are faced with interpreting vague symptoms unless an animal's status deteriorates quickly. Moreover, routine follow‐up procedures conducted after cardiac surgery in humans are not always scheduled in animals due to the need for sedation with each procedure. Finally, and most importantly, creating heart failure in the animal model may mask other complications when only non‐specific symptoms are present. Investigators tend to focus only on model changes. As in our case, when the pig showed tachycardia and laboured breathing, we first thought that chronic heart failure had been induced because it was a chronic ischemic model; this assumption caused a delay in diagnosis and treatment. Because echocardiography‐guided pericardiocentesis can save lives, increasing awareness of these possible complications after surgical intervention is important. All of these factors may contribute to the unnecessarily high mortality in large animal models, which increases costs, delays research progress, and does not optimise animal welfare.

Investigators could use new techniques to avoid postoperative complications and improve large animal care. For example, implanted telemetry is a powerful tool that can provide continuous EKG and blood pressure readings as well as other important data, which would allow investigators to make better informed medical decisions regarding animal care. Treating large animals should involve a similar level of care as given to human patients, with a professional care team and increased awareness of potential complications. For example, our introduction of a clinical anti‐arrhythmic protocol to the acute myocardial infarction pig model reduced mortality from 30% to 4.5% (Li et al., 2021).

In conclusion, we present here a rare case of late cardiac tamponade in a pig almost 30 days after cardiac surgery—a case that was entirely preventable. With the rapid development of large animal models, we should pay greater attention to improving animal care to decrease mortality rates, which would also ultimately benefit research progress.

## CONFLICT OF INTEREST

The authors declare no conflict of interest.

## AUTHOR CONTRIBUTIONS

Ke Li drafted the manuscript and performed echocardiography and animal care. Ana Maria Segura performed pathology studies. Junping Sun and Qi Chen designed the study and performed animal care. Jie Cheng designed the study and performed surgical procedures. Emerson C. Perin performed surgical procedures. Abdelmotagaly Elgalad drafted and critically reviewed the manuscript and performed surgical procedures and animal care. All authors read and approved the final version of the manuscript.

## FUNDING INFORMATION

The authors received no specific funding for this work.

## ETHICS STATEMENT

All surgical and animal care protocols used in this study were approved by the institutional animal care and use committee at our institution.

### PEER REVIEW

The peer review history for this article is available at https://publons.com/publon/10.1002/vms3.892

## Data Availability

The data that support the findings of this study are available upon request from the corresponding author.
